# The Future of Health Care: Going to the Dogs?

**DOI:** 10.3389/fneur.2015.00087

**Published:** 2015-05-08

**Authors:** William D. Freeman, Kenneth A. Vatz

**Affiliations:** ^1^Department of Neurology, Mayo Clinic, Jacksonville, FL, USA; ^2^Department of Neurosurgery, Mayo Clinic, Jacksonville, FL, USA; ^3^Department of Critical Care, Mayo Clinic, Jacksonville, FL, USA; ^4^CommunityHealth, Chicago, IL, USA

**Keywords:** healthcare costs, non-human employees, nosocomial infections, *C. difficile*, epilepsy, non-epileptic seizures, canines, animal rights

Escalating healthcare expenditures, which already represent 16% of the US’ gross domestic product (GDP), necessitate redesign of contemporary health care delivery modalities. One such model uses non-human employees (NHE) such as the *Canis domesticus*, which can potentially lower costs, provide sensitive detection of nosocomial pathogens, alert physicians and other caregivers to impending seizures, and provide comfort to patients. We propose consideration of canine NHE for appropriate clinical situations, but acknowledge the various limitations and caveats.

## Perspective

“Outside of a dog, a book is a man’s best friend. Inside of a dog it’s too dark to read.”

–Groucho Marx.

**Figure 1:**
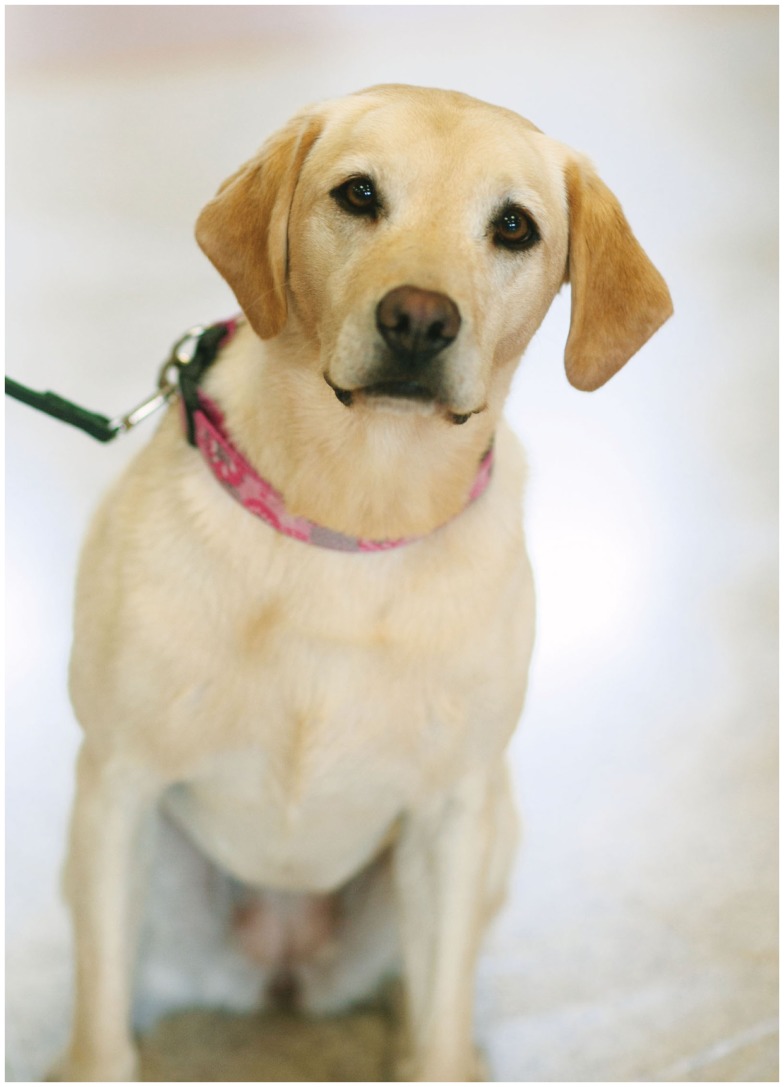
**The figure displays a potential candidate for an NHE program involved in the Mayo “Caring canines” program, which is a service in which canines visit hospitalized patients for psychological well-being**. This dog is calm, obedient, and well-mannered. While this candidate is a yellow Labrador, canine NHE are available in different breeds, sizes, and temperaments.

Healthcare expenditures comprise approximately 16% of the US GDP (1). Escalating costs have necessitated the redesign of contemporary health care delivery models in order to drive down costs and provide high-quality care. We propose one such model, which is utilizing “non-human employees” in healthcare. One indisputable example of underutilized NHE is the *C. domesticus*, colloquially referred to as “man’s best friend.” Canine NHE can be relatively low-cost alternatives to human employees (HE), with an initial capital expense on the order of $500–$2000 US [although the cost of obtaining a service dog can be as much as $30,000 US (2)], and between $1100 and $3500 US annually in maintenance (Figure 1) (3). Canine NHE may be more cost-effective when rescued from humane societies or other local repositories, and arguably constitute a tax-deductible expense for health care businesses.

At first blush, skeptics might have doubts regarding existing evidence supporting a canine NHE model. There is, in fact, considerable literature validating canine NHE use in healthcare. First, canines have been shown to be effective in detecting harmful hospital pathogens such a *Clostridium difficile* infection (4, 5), which can add considerable length of stay and cost for treating the disorder. Canine olfactory power is estimated to be up to 6 million times stronger than the human nose, thereby adding a tremendous biologic sensor in hospitals to detect *C. difficile* toxin (4, 5). *C. difficile* infection is an important nosocomial pathogen and a common cause of hospital diarrhea. The costs for management of *C. difficile* infection are estimated at around $800 million in USA and €3000 million in Europe annually (5). Second, canine counterparts may improve the physical and psychological health of humans, especially those with disabilities (6, 7). Third, canines may play a role in the detection of certain health problems in humans, including cancer, epileptic seizures (ES), and hypoglycemia (6). Even the Journal of the American Medical Association (JAMA) published an issue with cover artwork (*Dogs Playing Doctor*) paying homage to the early twentieth century artist Cassius Marcellus Coolidge (8).

Canine NHE have been proposed for the detection of ES (9). The role, however, for their detection remains uncertain, and the tendency to identify non-epileptic seizures (NES) during video-electroencephalographic (EEG) monitoring has been a confounding factor (10, 11). To date, the results have been mixed in terms of sensitivity and specificity to both patients with ES and NES and regarding the ability of canine NHE to distinguish between the two types of events. Regardless of the pathophysiology, NHE also provide a degree of psychological comfort to patients (11–13).

Therefore, we propose that canine NHE be considered in future health care delivery models as a potential value added measure, even considering the additional ongoing expenses of the consistent human companionship required for canines working in these roles. For many of these functions, such as prescribing and administering medications, canines still cannot replace human care givers. It must be emphasized, however, that the benefits of canine NHE go beyond the mechanics of care giving, in that they unquestionably provide companionship and much emotional support for both human health care employees and patients. Of course, as NHE become more prevalent, animal rights groups are expected to advocate for such canines to seek legal “personhood” similar to that of chimpanzees (14), thus potentially reducing their adoption into health care use due to increased overhead and administrative costs.

## Conflict of Interest Statement

The authors declare that the research was conducted in the absence of any commercial or financial relationships that could be construed as a potential conflict of interest.
